# Maternal health literacy plays a greater role than paternal health literacy in adolescent physical activity in China: a cross-sectional study

**DOI:** 10.3389/fpubh.2025.1585615

**Published:** 2025-05-19

**Authors:** Nanfang Yu

**Affiliations:** School of Sports Science, Qufu Normal University, Qufu, Shandong, China

**Keywords:** adolescent, health literacy, physical activity, obesity, lifestyle

## Abstract

**Background:**

Given the prevalence of obesity and physical inactivity among Chinese adolescents, assessing the role of parents in this situation is important. Therefore, the main aim of this study was to evaluate the association between parental health literacy levels and adolescents' physical activity and obesity in China.

**Methods:**

In this cross-sectional study, a sample of 4,717 Chinese high school students and their parents participated. Parental health literacy was measured using the Chinese version of the Health Literacy Survey 2019-12-item questionnaire (HLS19-Q12), and adolescents' physical activity was assessed using the short version of the International Physical Activity Questionnaire (IPAQ). Body mass index (BMI) was calculated using standardized measurements of weight and height (kg/m^2^). Statistical analyses, including independent *t-*test and multiple linear regression (to examine predictors of adolescent physical activity and BMI) were performed using SPSS 26.0 software. The significance level was set at *p* < 0.05.

**Results:**

A total of 31.95% of adolescents were overweight or obese, and 48.15% had low physical activity, which was significantly lower in girls than in boys (*p* = 0.01, Cohen's *d* = 0.212). Only 21.39% of parents had excellent health literacy. Higher maternal health literacy was significantly associated with increased physical activity in boys, whereas both maternal and paternal health literacy were associated with higher physical activity in girls, with maternal health literacy showing a stronger association than paternal health literacy (*p* < 0.001). Furthermore, low physical activity was the only factor significantly associated with higher BMI among students (*p* < 0.001).

**Conclusion:**

The findings highlight the importance of addressing the health literacy of parents, especially mothers, in promoting adolescent physical activity and preventing obesity. Health promotion programs and educational policies should consider family-based approaches to effectively address gender-specific needs and improve adolescent health outcomes.

## 1 Introduction

As a country experiencing rapid economic growth and extensive social changes, China is witnessing notable shifts in the lifestyle patterns of adolescents. Obesity is growing concern among adolescents worldwide, including in China (1, 2). In recent decades, the prevalence of obesity in this age group has increased significantly, posing serious risks to both physical and mental health (3, 4). Studies show that since the 1980s, obesity rates among Chinese children and adolescents have steadily increased, making China one of the countries with the highest obesity rates in Asia (3, 4). In 2016, 12.9% of Chinese adolescents were overweight or obese, a significant rise compared to previous years (3). Research also indicates that two out of every five Chinese children and adolescents are overweight or obese, with estimates suggesting this number could reach 60% by 2030 (4).

Key contributors to this trend include lifestyle changes, greater consumption of processed foods, reduced physical activity levels, and increased sedentary behaviors such as prolonged use of digital devices. Several studies have linked decreased physical activity and increased screen time among Chinese adolescents to rising obesity rates (5, 6).

Adolescent lifestyle is shaped by individual, family, and environmental factors. Among these, the family may plays a central role in shaping children's health-related behavioral (7). Some studies in China have assessed the association of family factors with adolescent health behaviors. For example, a study from Cai et al. (8) found that adolescents' nutritional knowledge was associated with a reduced risk of overweight and obesity, while parental nutritional knowledge had no significant effect. The family is considered the most important social institution in Chinese culture, and family values have a strong influence on adolescents' health and lifestyle decisions (9). In this culture, parents usually have the primary responsibility for educating and guiding their children and monitoring their health behaviors. Studies show that families in China play a particularly key role in the nutrition and physical activity of adolescents, as many decisions related to diet and physical activity are influenced by parental behavioral patterns (9). These behavioral patterns can significantly influence lifestyle and health choices, especially during adolescence when individuals are influenced by social and cultural norms. For example, Chinese parents tend to encourage their children to be more physically active, but at the same time, cultural and social changes in China can lead to a decrease in physical activity and an increase in inactive behaviors such as the use of digital devices (10). These social and cultural changes may pose new challenges for promoting health behaviors in adolescents. Therefore, a deeper understanding of how culture and family influence adolescent health behaviors in China may help develop appropriate health programs and interventions to promote healthy lifestyles in this population.

Parents can provide a supportive environment that promotes healthy lifestyle, influencing their children's physical activity, nutrition status, and body composition. Thus, improving parental awareness through education and access to reliable information can enhance health literacy, reduce diseases risk, improve quality of life, and support healthier family lifestyle (11).

Health literacy refers to the ability of person to access, understand, evaluate, and use health-related information to make informed decision (12). Research shows that the higher health literacy is associated with healthier behaviors and a reduced risk of disease (13). Given the key role of parents in shaping children's behaviors, understanding how parental health literacy affects adolescent behaviors—particularly physical activity—is crucial. Some evidence suggests that parental health literacy can play a critical role in shaping children's health outcomes. Parents with higher health literacy are better able to understand nutritional guidelines, promote active lifestyles, and limit sedentary behaviors such as excessive screen use in their children (14, 15). Furthermore, parents with high health literacy are more able to make informed decisions about health and act as positive role models, influencing their children's behavior through direct guidance and behavioral modeling (16). Some studies have also highlighted the relationship between low parental health literacy and increased risk of obesity and overweight in children (17).

Despite a growing body of literature on adolescent health behaviors, few studies have examined the association between parental health literacy and adolescents' physical activity and lifestyle choices in the context of Chinese families. Some scientific gaps remain in understanding how parental health literacy influences adolescent physical health. Several factors such as gender, age, and socioeconomic status may moderate this association (18). In China, where adolescent lifestyles are rapidly changing, and obesity rates are rising, exploring this association is especially importance. This study assesses how parental health literacy influences physical activity and obesity rates in adolescents in China. The findings of this study contribute to a new understanding of the role of health literacy in shaping adolescent behaviors and provide practical implications for the development of family-based interventions and policy strategies to promote healthy lifestyles in adolescents. This research also contributes to the growth of the health literacy field, focusing on Chinese adolescents who face specific cultural and social challenges related to obesity and inactivity.

## 2 Materials and methods

### 2.1 Study design and participants

This cross-sectional study was conducted from August to November 2024 in Shandong Province, China. To ensure socioeconomic diversity, all districts in the province were first grouped into three levels—high, medium, and low economic development—based on indexes such as urbanization rate, average household income, and per capita GDP. These data were collected from Shandong Provincial Bureau of Statistics (19). Then, one district was selected from each economic category using a simple random sampling method, representing a total of three districts with different levels of economic development. From each selected district, one public senior secondary school was randomly selected from an official list provided by the local education authorities. In the Chinese education system, secondary education is divided into junior (grades 7–9) and senior (grades 10–12) levels. Since part of the questionnaire required input from parents, participation in the study required informed consent from both students and at least one of their parents. Both parents were invited to complete the questionnaire. For each student, parental data included responses from one or both parents depending on participation. Therefore, data were analyzed based on the data provided: in cases where only one parent responded, that parent's data was used; in cases where both parents responded, both sets of data were included in the analysis. For 44.98% of adolescents, data were collected from both parents.

Therefore, inclusion criteria were: enrollment as a full-time student in one of the selected public senior secondary schools, being between 16 and 18 years old, willingness to participate, and participation of at least one parent, including completion of the relevant portion of the questionnaire and provision of written informed consent. Exclusion criteria included: presence of any diagnosed medical condition that precluded participation in physical activity (e.g., chronic illness), incomplete or inconsistent responses in the questionnaire (from either the student or the parent), and enrollment in a private school.

Written informed consent was obtained from participating parents. To ensure the quality of reporting, this study adhered to the Strengthening the Reporting of Observational Studies in Epidemiology (STROBE) guidelines for cross-sectional study (20). Ethical approval for this study was approved by the Research Ethics Committee of Shandong Provincial Center for Disease Control and Prevention (NO: SHCDC-202406).

### 2.2 Sample size

The sample size was estimated using G-power (version 3.1). Based on an expected medium effect size (*d* = 0.5), statistical power of 0.90, and a significance level of α = 0.05, the calculated minimum sample size was 3,498. To account for an anticipated 15% dropout rate, a total of 4,023 participants were targeted for recruitment. A statistical power of 0.90 was selected for calculating sample size to provide greater sensitivity in detecting even small to medium effects, which is particularly important in behavioral and public health research. According to Faul et al. (21), a power of 0.90 is generally recommended when researchers want to reduce the risk of type II errors and increase the likelihood of detecting significant differences, especially in studies expected to have medium effects. Also, the effect size was selected in accordance with Cohen's guidelines for behavioral studies (22).

### 2.3 Measurements

One section of questionnaire gathered socio-demographic information about adolescents and their parents, including gender (male/female), age, parental educational level (less than high school, high school diploma, and above diploma), and household annual income (20,000 CNY or below, 20,000-39,000 CNY, 40,000–59,000 CNY, 60,000–79,000 CNY, 80,000–149,000 CNY, and 150,000 CNY or above).

In another section, after measuring the weight and height of the adolescents at school, BMI was calculated as weight (kg) divided by height squared (m^2^). Standardized techniques and equipment were used for measurements. We used three parameters age, gender, and BMI to assess overweight and obesity among students. The classification was based on the 2005 China national BMI growth charts and reference data for children and adolescence (23). According to this guideline, adolescents were categorized as follows: normal weight (BMI < 85th percentile for age), overweight (85th−94.9th percentile), and obese (BMI ≥ 95th percentile) (23). This measurement method has also been used in other Chinese studies (24–26).

Parental health literacy was assessed using the Chinese version of the HLS19-Q12. This scale comprises three health domains, including health care, disease prevention, health promotion, as well as four competencies of health information processing, including accessing, understanding, evaluating, and applying health information (27). Each item was rated on a four-point Likert scale (1 = very difficult to 4 = very easy), reflecting the perceived difficulty of each task. The total score was calculated as a percentage (ranging from 0 to 100) of items with valid responses that were rated as “very easy” or “easy” provided that at least 80% of the items had valid response. If fewer than 80% of the items had valid responses, the score was coded as “missing.” Higher HLS19-Q12 scores indicate higher levels of health literacy. A categorical variable was also calculated to reflect the population distribution of health literacy levels. Health literacy was categorized into four categories: (1) Excellent: More than half of the items were answered as “very easy,” and no more than one item was rated as “very difficult” or “difficult.”; (2) Sufficient: At least 10 out of 12 items were rated as “very easy” or “easy,” and no more than two items were rated as “very difficult” or “difficult.”; (3) Problematic: Respondents who did not meet the criteria for the “excellent,” “sufficient,” or “inadequate” categories; and (4) More than half of the items were rated as “very difficult” or “difficult,” and no more than one item was rated as “very easy” (27). The Chinese version of this questionnaire has demonstrated good psychometric properties in previous validation studies (28). In the current study, internal consistency was good, with a Cronbach's alpha of 0.83, indicating satisfactory reliability.

Physical activity of adolescents was assessed using the short form of the International Physical Activity Questionnaire (IPAQ-SF) (29), which has been previously validated among Chinese youth populations (30). In this study, the internal consistency of the IPAQ-SF was acceptable (Cronbach's alpha = 0.71). The questionnaire evaluates the frequency (days per week) and duration (minutes per day) of walking, moderate-intensity activity, and vigorous-intensity activity (31, 32). Each activity is assigned a metabolic equivalent (MET): walking= 3.3 METs, moderate activity = 4 METs, and vigorous activity = 8 METs (33). To calculate the MET score for each physical activity, the MET value is multiplied by the total minutes of that physical activity performed per week. The total physical activity MET score is obtained by summing the MET values of all reported activities. Based on the total MET score, participants were categorized into three levels of physical activity (34, 35): low physical activity: <850 METs/week, moderate physical activity: 851–1,500 METs/week, high physical activity: >1,500 METs/week.

### 2.4 Statistical analyses

Quantitative data were presented as mean ± standard deviation (SD), while qualitative data were reported as frequencies and percentage. An independent *t-*test was utilized to compare gender differences (among adolescents and parents) across selected variables. To identify the predictors of adolescents' physical activity and BMI, multiple linear regression analysis was conducted. No multicollinearity was detected among the independent variables (tolerance > 0.1 and variance inflation factor or VIF < 10). Effect sizes for *t-*tests were calculated using Cohen's *d*. All statistical analysis was performed using SPSS version 26.0. The significance level was set at *p* < 0.05.

## 3 Results

**Figure 1:**
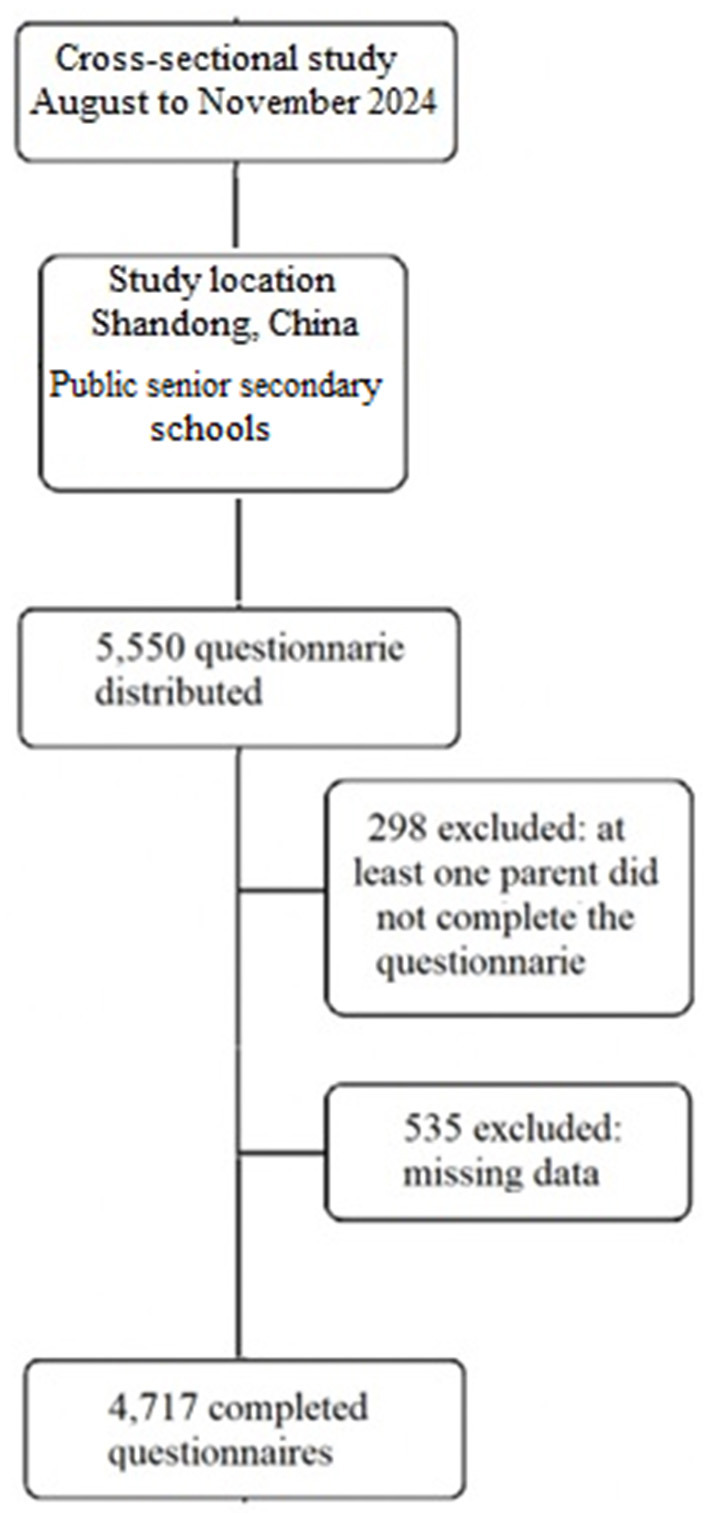
Flow diagram of participant recruitment and analysis.

A total of 5,550 questionnaires were distributed; of which 4,717 were included in the final statistical analysis (response rate = 85.02%) (Figure 1). Descriptive characteristics of participants are presented in Table 1. The adolescents had a mean age of 16.8 ± 0.83 years, and 50.98% were girls. The mean BMI was 22.34 ± 4.21 kg/m^2^, with an overall prevalence of overweight and obesity of 31.95%. The mean physical activity level was 877.47 ± 539.67 METs/week. Based on the predefined criteria, 48.15% of adolescents were classified as having low physical activity. Regarding gender differences, girls had significantly lower physical activity levels than boys (752.97 ± 461.39 in girls vs. 1,001.98 ± 617.95 in boys, *p* = 0.01, and boys had significantly higher BMI compared to girls (22.82 ± 4.81 in boys and 21.86 ± 3.61 in girls, *p* = 0.001) (Table 1). The effect sizes of both differences were small (*d* = 0.212 and 0.233 for physical activity and BMI).

**Table 1:** Participant characteristics.

**Participants**	**Variables**		**All**	**Male**	**Female**	***P***
Adolescents	Age (year)	Mean ± SD	16.8 ± 0.73	16.61 ± 0.9	16.99 ± 0.76	0.42
	BMI (kg/m2)	Mean ± SD	22.34 ± 4.21	22.82 ± 4.81	21.86 ± 3.61	0 < 001
		Normal	68.05% (3,209)	65.8% (1,522)	70.2% (1,689)	
		Overweight	22.97% (1,084)	24.47% (566)	24.44% (588)	
		Obesity	8.98% (424)	9.73% (225)	5.36% (199)	
	Physical activity (MET/week)	Mean ± SD	877.47 ± 539.67	1001.98 ± 617.95	752.97 ± 461.39	0.01
		Low	48.15% (2,271)	41.59% (962)	55% (1,310)	
		Moderate	30.98% (1,461)	32% (740)	30% (722)	
		High	20.87% (985)	26.41% (611)	15% (374)	
Parents	Age (year)	Mean ± SD	39.21 ± 3.29	38.45 ± 3.27	39.97 ± 3.31	0.512
	Educational level	Less than high school	21.1% (996)	17.44% (391)	24.5% (605)	
		High school diploma	53% (2,501)	54.68% (1,226)	51.47% (1,275)	
		Above diploma	25.9% (1,222)	27.88% (625)	24.10% (597)	
	Household annual income (CNY)	20,000 or below	8.3% (393)			
		20,000 to 39,000	10.6% (501)			
		40,000 to 59,000	29.8% (1402)			
		60,000 to 79,000	28.42% (1341)			
		80,000 to 149,000	13.58% (643)			
		150,000 or above	9.3% (439)			
	HLS-Q12	Mean ± SD	82.34 ± 22.45	82.49 ± 21.61	82.19 ± 23.29	0.121
		Excellent	21.39% (1,009)	21.14% (474)	21.6% (535)	
		Sufficient	49.5% (2,336)	47.99% (1,076)	50.87% (1,260)	
		Problematic	22.2 (1,049)	22.26% (499)	22.2% (550)	
		Inadequate	6.89% (325)	8.61% (193)	5.33% (132)	

Students: 4,717: 2,313 males and 2,404 females; Parents: 4,719: 2242 males and 2,477 females; BMI, Body Mass Index; MET, Metabolic Equivalent of Task; CNY, Chinese Yuan; HLS-Q12, Health Literacy Survey 2019-12-item questionnaire; An independent t-test was utilized to compare gender differences.

Parents had a mean age of 39.21 ± 3.29 years, with females comprising 52.5% of the sample. 25.9% of participating parents had education levels above diploma and 9.3% reported a household income of 150,000 CNY or above. The mean parental health literacy score was 82.34 ± 22.45, with no significant difference between mothers and fathers (*p* > 0.05). Based on the health literacy classification, 21.39% of parents had excellent health literacy, while 6.89% were in the inadequate category (Table 1).

In this study, multiple linear regression models were used to identify the factors influencing students' physical activity levels. The results indicated that the overall model was statistically significant (*F* = 17.93, *p* < 0.001), suggesting that at least one of the independent variables significantly predicted the dependent variable. Also, the model was able to predict 13.8% of the variance in adolescents' physical activity. Among the predictors, maternal health literacy (β = 0.334, *p* < 0.001), paternal health literacy (β = 0.219, *p* < 0.001), and student's gender (being male) (β = 0.120, *p* < 0.001) were found to significantly increased physical activity in adolescents. The beta coefficients suggest that maternal health literacy had the strongest predictive power, followed by gender and then paternal health literacy.

When analyzing the data by gender, and after controlling for gender-related effects, maternal health literacy remained a significant predictor of physical activity among boys (β = 0.301, *p* < 0.001). For girls, both maternal (β = 0.289, *p* < 0.001) and paternal health literacy (β = 0.215, *p* < 0.001) significantly predicted physical activity, with maternal health literacy showing a greater impact than paternal health literacy (Table 2).

**Table 2:** Multiple linear regression model predicting adolescents' physical activity levels.

**Model^a^**		**All**		**Males**		**Females**	
		**Unstandardized**	**Standardized**	**Unstandardized**	**Standardized**	**Unstandardized**	**Standardized**
(Constant)		29.13		28.41		29.02	
Parents' age		0.010	0.003	0.020	0.02	0.018	0.003
Adolescents gender	Female						
	Male	0.075^**^	0.120				
Maternal' educational level	Under diploma						
	Diploma	0.014	0.012	0.011	0.013	0.015	0.011
	Above diploma	0.023	0.013	0.021	0.016	0.018	0.013
Paternal' educational level	Under diploma						
	Diploma	0.017	0.016	0.015	0.015	0.016	0.014
	Above diploma	0.025	0.020	0.023	0.018	0.021	0.015
Annual income	20,000 or below						
	20,000–39,000	0.018	0.001	0.020	0.010	0.014	0.003
	40,000–59,000	0.01	0.006	0.019	0.002	0.018	0.014
	60,000–79,000	0.020	0.02	0.020	0.020	0.02	0.004
	80,000–149,000	0.022	0.015	0.023	0.010	0.018	0.007
	150,000 or above	0.029	0.014	0.025	0.011	0.017	0.02
Father health literacy		0.081^**^	0.219	0.035	0.225	0.072^**^	0.215
Mother health literacy		0.091^**^	0.333	0.073^**^	0.301	0.086^**^	0.289
		*F =* 17.93, *p <* 0.05, adjusted R^2^= 0.138		*F =* 16.41, *p <* 0.05, adjusted R^2^= 0.128		*F =* 17.77, *p <* 0.05, adjusted R^2^= 0.111	

a Dependent variable: physical activity;

** 0 < 0.001.

A multiple linear regression model was also used to identify predictors of students' BMI (Table 3). The model results indicated that at least one independent variable significantly predicted BMI (*F* = 16.21, *p* < 0.001). The model accounted for 9.6% of the variance in BMI. Across the overall sample and in both gender subgroups, only low physical activity significantly predicted higher BMI scores (β = −0.411, *p* < 0.001).

**Table 3:** Predicting adolescents' BMI using multiple linear regression.

**Model^a^**		**All**		**Males**		**Females**	
		**Unstandardized**	**Standardized**	**Unstandardized**	**Standardized**	**Unstandardized**	**Standardized**
(Constant)		20.45		19.71		20.65	
Parents' age		0.003	0.09	0.031	0.011	0.026	0.039
Adolescents' Gender	Female						
	Male	0.015	0.08				
Maternal' educational level	Under diploma						
	Diploma	0.09	0.099	0.018	0.010	0.007	0.023
	Above diploma	0.011	0.010	0.017	0.022	0.008	0.012
Paternal' educational level	Under diploma						
	Diploma	0.10	0.086	0.015	0.008	0.009	0.021
	Above diploma	0.013	0.009	0.009	0.020	0.011	0.014
Annual income	20,000 or below						
	20,000–39,000	0.014	0.011	0.017	0.09	0.010	0.014
	40,000–59,000	0.007	0.088	0.010	0.031	0.004	0.02
	60,000–79,000	0.012	0.087	0.015	0.012	0.011	0.09
	80,000–149,000	0.008	0.09	0.011	0.007	0.003	0.090
	150,000 or above	0.010	0.016	0.011	0.010	0.001	0.099
Father health literacy		0.011	−0.09	0.003	0.007	0.010	0.015
Mother health literacy		0.015	−0.091	0.011	0.010	0.008	0.10
Adolescents' physical activity		0.091^**^	−0.411	0.085^**^	−0.210	0.083^**^	−0.225
		*F =* 16.21, *p <* 0.05, adjusted R^2^= 0.096		*F =* 15.10, *p <* 0.05, adjusted R^2^= 0.085		*F =* 15.28, *p <* 0.05, adjusted R^2^= 0.069	

a Dependent variable: BMI;

** 0 < 0.001.

## 4 Discussion

The findings of this study revealed that the prevalence of overweight and obesity among adolescents aged 16–18 years was 31.95%. While the prevalence of overweight and obesity among children and adolescents aged 0–19 years in China was significantly lower than the global average (8.8%) in 2000, there has been a dramatic increase of nearly 400% over the past two decades, reaching an estimated 37.9% in 2020. This figure surpasses the rates reported in other Western Pacific sub-regions and upper-middle-income countries (4). Current projections estimate that, by 2030, ~60% of Chinese children and adolescents will be affected by overweight or obesity (4). This upward trend may be attributed to unhealthy lifestyle behaviors, including increased consumption of high-calorie foods and reduced physical activity (36). In the present study, ~48% of adolescents were categorized as physically inactive, a known key factor increasing to the development of overweight and obesity (36). Some studies have reported a higher prevalence. For instance, a study by Lee et al. showed that more than half of Chinese adolescents did not meet recommended physical activity (37). Another study by Zhang et al. reported that 71.4% of Chinese children and adolescents in their sample were physically inactive (38). Variations in the reported prevalence rates across studies may be attributed to differences in age groups of participants, measurement tools, operational definitions of physical inactivity.

The findings of this study indicated that Chinese parents' health literacy significantly predicted the physical activity levels of their adolescent children. To the best of our knowledge, no previous study has directly examined the association between parental health literacy and adolescents' physical activity levels in the Chinese context. A study conducted in Germany found that higher parental health literacy was associated with more physically active children and adolescents (19). Also, Lee et al. (39) found that higher parental functional health literacy significantly predicted better health outcomes in children aged 4–10 years (20).

Parental health literacy may influence adolescent physical activity through several mechanisms. One possible pathway is through modeling, in which parents with higher health literacy are more likely to engage in physical activity themselves, acting as positive role models for their children. Adolescents, especially boys, tend to imitate their parents' behaviors, which can lead to increased physical activity. In addition, emotional support from health-literate parents, such as encouragement and positive reinforcement, plays a key role in motivating adolescents to engage in physical activity (17). Another factor that may be important is awareness of resources. Parents with higher health literacy are more likely to be aware of community resources, such as local sports programs, clubs, and parks, and are more likely to encourage their children to use these resources. Access to these resources makes adolescents more likely to participate in physical activity (40). Parental communication styles also play an important role. Parents with high health literacy are more likely to use positive and supportive communication styles about the importance of physical activity, help their children set achievable goals for physical activity, and provide positive feedback. This effective communication leads to adolescents having more positive attitudes toward physical activity and being more motivated to do so (17).

Specifically, maternal health literacy was associated with increased physical activity among boys, whereas that health literacy of both parents was associated with increased physical activity among girls. To our knowledge, no prior study has specifically evaluated the differential effects of maternal and paternal health literacy on adolescents' physical activity levels. However, existing evidence suggests that mothers tend to provide greater support for their sons' physical activity (41). Additionally, research indicates that mothers are often more involved in the organizational aspects of their children's physical activity, while fathers are more likely to serve as role models for physical activity behavior (42). A study has shown that children, particularly boys, tend to form stronger emotional bonds with their mothers during childhood and adolescence. For this reason, they naturally look to their mothers as the main sources of security in their lives. Therefore, it is likely that maternal health literacy will enable them to pass on accurate information and guidance regarding physical activity and health to their children (43). Also, mothers play a more prominent role in socializing their children's physical activity (44). Mothers with high health literacy are likely to play a key role in organizing physical activity and encouraging their children to participate in it. A study has shown that mothers influence their children's lifestyle decisions by teaching them emotion regulation skills (45). The impact of mothers on adolescent, especially boys, can be understood as a combination of psychological, social, and biological factors. Through secure attachment, stress reduction, enhancement of social skills, cognitive development, and cultural values transmission, mothers can exert a profound and lasting influence on adolescent development. This influence extends beyond adolescence, shaping decision-making processes and the formation of healthy social relationships well into adulthood (46).

In the present study, gender was found to be a significant predictor of adolescents' physical activity levels, with boys exhibiting higher levels of physical activity than girls. This finding is consistent with previous research reporting higher physical activity among Chinese boys compared to girls (47, 48). In many societies, including China, gender roles and expectations play a substantial role in shaping physical activity participation. Boys are often more encouraged to engage in sports and physical activities, whereas girls may face social constraints or expectations that limit their participation (49). Additionally, boys tend to participate more in school- based sports and have greater access to sports facilities. In contrast, girls are often less likely to engage in sports due to social pressures or concerns related physical appearance (50). From a biological perspective, hormonal differences may also contribute to these disparities. During adolescence, elevated testosterone levels in boys lead to increased muscle mass and a greater tendency toward vigorous physical activities (51).

In relation to BMI, the findings of this study revealed that only high physical activity levels were significant predictors of lower BMI among Chinese adolescents. This association between physical activity and lower BMI has been consistently documented across different populations, including China. For instance, a study investigating patterns of physical activity and BMI among Chinese adolescents found that those who engaged in higher levels of physical activity had significantly lower BMI scores (52). These results underscore the beneficial role of physical activity in weight control and body composition during adolescence. Furthermore, another study demonstrated that increased physical activity was associated with a reduced risk of overweight and obesity in Chinese adolescents (53). These findings highlight the critical need to promote regular physical activity through school-based and community-level programs to support adolescent health and prevent obesity. Engaging in regular physical activity contributes to energy balance and reduces fat accumulation by increasing energy expenditure and metabolism efficiency. Additionally, exercise improves body composition by increasing muscle mass and decreasing fat mass, thereby resulting in a lower BMI (54).

This study, while offering valuable findings, has several limitations that should be acknowledged when interpreting the findings. First, the cross-sectional design poses a limitation in inferring causal inferences; therefore, future studies employing longitudinal designs is recommended to explore the long-term effects of parental health literacy on adolescent' physical activity. Second, physical activity levels were assessed using self-report data. Participants may have overestimated their physical activity levels, especially given that the questionnaire was completed in a school setting, increasing the likelihood of prosocial responses. This overreporting could lead to an overestimation of the proportion of adolescents who are at moderate or high levels of physical activity. Such biases may also have weakened or strengthened the observed associations between physical activity and parental health literacy, particularly if the extent of reporting error was related to parental characteristics. Therefore, caution should be exercised when interpreting the prevalence estimates as well as the strength of associations related to physical activity in this study. The incorporation of objective measurement instruments, such as accelerometers and wearable activity trackers in future studies could enhance the accuracy of physical activity assessment. Third, data were collected from only one province (Shandong); therefore, the generalizability of the findings to other regions in China with different economic characteristics, urbanization level, education system, and cultural attitudes toward parental involvement and adolescent health behaviors may be limited. Consequently, future studies in more geographically diverse areas could help validate and extend the findings of this study. Also, this study did not collect data on household size, which can play an important role in family dynamics, the amount of parental supervision, and adolescents' opportunities for physical activity. In addition, as all participants were recruited from urban schools, the study results may not be generalizable to adolescents living in rural areas, as access to physical activity resources and patterns of parental involvement may differ in these areas. These limitations should be considered in the overall interpretation of the study results.

One limitation of this study was that it did not account for the clustering effects at the school level. Participants attended three different schools, and school characteristics (such as health policies, quality of health education, or socioeconomic status of the school environment) may have influenced study outcomes. Failure to model these effects as random effects may have resulted in an underestimation of the standard error, thereby increasing the likelihood of a type I error (finding a relationship where none actually exists). Therefore, the findings may have been reported with higher statistical confidence than they actually were. In future studies, it is recommended to use multilevel modeling approaches to better account for hierarchical data structures and improve the precision of estimates.

One limitation of this study was the lack of examination of students from non-government schools, which may reduce the generalizability of the results to the entire adolescent population.

## 5 Conclusion

The findings of this study highlight the critical role of parents in promoting adolescent physical activity and demonstrate the direct influence of parental health literacy on their children's physical activity. Accordingly, public health initiatives and educational programs should not only focus on adolescents but also activity involve parents in fostering a more active lifestyle within families by enhancing health literacy. Specifically, attention to maternal health literacy and supporting mothers can have a great impact on increasing physical activity, particularly among boys. In addition, efforts should be directed at addressing gender inequalities in physical activity, so that girls and boys are provided with equal opportunities and support in all educational and social contexts. These efforts can be strengthened by designing educational programs and public policies that focus on promoting parental health literacy and gender equality in access to physical activity. Finally, the findings of this study can serve as a basis for public health policies to address adolescent obesity and physical inactivity in China. Developing and implementing educational programs at the family and community levels can have long-term effects in preventing health problems associated with physical inactivity and obesity in adolescents.

## Data Availability

The original contributions presented in the study are included in the article/Supplementary material, further inquiries can be directed to the corresponding author.

## References

[1] Hadier SG Yinghai L Long L Hamdani SD Hamdani SMZH. Mediation role of cardiorespiratory fitness on association of physical activity and physical literacy among 8–12 years old children: the PAK-IPPL cross-sectional study. Front Pediatr. (2024) 12:1383670. 10.3389/fped.2024.1383670 39346638 PMC11427255

[2] Hadier SG Yinghai L Long L Hamdani SD Hamdani SMZH. Assessing physical literacy and establishing normative reference curves for 8–12-year-old children from South Punjab, Pakistan: The PAK-IPPL cross-sectional study. PLoS ONE. (2025) 20:e0312916. 10.1371/journal.pone.0312916 39932941 PMC11813120

[3] Pan X-. F Wang L Pan A. (2021). Epidemiology and determinants of obesity in China. Lancet Diabet Endocrinol. 9:373–92. 10.1016/S2213-8587(21)00045-0 34022156

[4] Okunogbe A Nugent R Spencer G Powis J Ralston J Wilding J. Economic impacts of overweight and obesity: current and future estimates for 161 countries. BMJ Global Health. (2022) 7:e009773. 10.1136/bmjgh-2022-009773 36130777 PMC9494015

[5] Nan J Chen M Yuan H Cai S Piao W Li F. Prevalence and influencing factors of central obesity among adults in China: China Nutrition and Health Surveillance (2015–2017). Nutrients. (2024) 16:2623. 10.3390/nu16162623 39203759 PMC11357308

[6] Huang Z Tian Z Cui J Wang G Chen J. Prevalence of overweight/obesity, and associated factors among adolescents aged 12–15 in Shandong Province, China: a cross-sectional study. Prevent Med Rep. (2024) 45:102831. 10.1016/j.pmedr.2024.102831 39193377 PMC11347837

[7] Tabrizi JS Doshmangir L Khoshmaram N Shakibazadeh E Abdolahi HM Khabiri R. Key factors affecting health promoting behaviors among adolescents: a scoping review. BMC Health Serv Res. (2024) 24:58. 10.1186/s12913-023-10510-x 38212786 PMC10782684

[8] Cai Z Jiang K Wang T Li S Xian J Zhao Y. Influence of adolescents' and parental dietary knowledge on adolescents' body mass index (BMI), overweight/obesity in 2004–2015: a longitudinal study. Arch Public Health. (2023) 81:188. 10.1186/s13690-023-01197-x 37872636 PMC10591379

[9] Liu KS Chen JY Ng MY Yeung MH Bedford LE Lam CL. How does the family influence adolescent eating habits in terms of knowledge, attitudes and practices? A global systematic review of qualitative studies. Nutrients. (2021) 13:3717. 10.3390/nu13113717 34835973 PMC8624651

[10] Wang L Qi J. Association between family structure and physical activity of Chinese adolescents. BioMed Res Int. (2016) 2016:4278682. 10.1155/2016/4278682 27123446 PMC4829685

[11] Arafa A Yasui Y Kokubo Y Kato Y Matsumoto C Teramoto M. Lifestyle behaviors of childhood and adolescence: contributing factors, health consequences, and potential interventions. Am J Lifestyle Med. (2024) 15598276241245941. 39554934 10.1177/15598276241245941 PMC11562273

[12] Ogbadu-Oladapo L Bissadu K Kim H Smith DL. Information and health literacy: could there be any impact on health decision-making among adults?—evidence from North America. J Public Health. (2024) 32:1–31. 10.1007/s10389-024-02260-9

[13] Fang S Mushtaque I. The moderating role of health literacy and health promoting behavior in the relationship among health anxiety, emotional regulation, and cyberchondria. Psychol Res Behav Manag. (2024) 51–62. 10.2147/PRBM.S446448 38196775 PMC10775698

[14] DeWalt DA Berkman ND Sheridan S Lohr KN Pignone MP. Literacy and health outcomes: a systematic review of the literature. J Gen Intern Med. (2004) 19:1228–39. 10.1111/j.1525-1497.2004.40153.x 15610334 PMC1492599

[15] Sanders LM Federico S Klass P Abrams MA Dreyer B. Literacy and child health: a systematic review. Arch Pediatr Adolesc Med. (2009) 163:131–40. 10.1001/archpediatrics.2008.539 19188645

[16] Manganello JA. Health literacy and adolescents: a framework and agenda for future research. Health Educ Res. (2008) 23:840–7. 10.1093/her/cym069 18024979

[17] de Buhr E Tannen A. Parental health literacy and health knowledge, behaviours and outcomes in children: a cross-sectional survey. BMC Public Health. (2020) 20:1–9. 10.1186/s12889-020-08881-5 32660459 PMC7359277

[18] Fleary SA Joseph PL. Health literacy and health behaviors in parent-adolescent dyads: an actor-partner interdependence model approach. Psychol Health. (2024) 39:803–22. 10.1080/08870446.2022.2117809 36047615 PMC10013691

[19] Jin Z Wang C Jiao X Yu S Yang C Xie F. Spatiotemporal pattern and influencing factors of urbanization quality in county areas of Shandong Province, China. Ecol Indic. (2024) 163:112132. 10.1016/j.ecolind.2024.112132

[20] Von Elm E Altman DG Egger M Pocock SJ Gøtzsche PC Vandenbroucke JP. The Strengthening the Reporting of Observational Studies in Epidemiology (STROBE) statement: guidelines for reporting observational studies. Lancet. (2007) 370:1453–7. 10.1016/S0140-6736(07)61602-X 18064739

[21] Faul F Erdfelder E Buchner A Lang A-G. Statistical power analyses using G^*^ Power 3.1: Tests for correlation and regression analyses. Behav Res Meth. (2009) 41:1149–60. 10.3758/BRM.41.4.1149 19897823

[22] Cohen J. Statistical power analysis for the behavioral sciences. Milton Park: Routledge (2013).

[23] Li H Ji CY Zong XN Zhang YQ. Body mass index growth curves for Chinese children and adolescents aged 0 to 18 years. Chin J Pediat. (2009) 47:493–8. 19951508

[24] Li H Xiang X Yi Y Yan B Yi L Ding N. Epidemiology of obesity and influential factors in China: a multicenter cross-sectional study of children and adolescents. BMC Pediatr. (2024) 24:498. 10.1186/s12887-024-05209-9 39095721 PMC11295318

[25] Song P Li X Gasevic D Flores AB Yu Z. BMI, waist circumference reference values for Chinese school-aged children and adolescents. Int J Environ Res Public Health. (2016) 13:589. 10.3390/ijerph13060589 27314368 PMC4924046

[26] Dong Y Ma J Song Y Dong B Wang Z Yang Z. National blood pressure reference for Chinese Han children and adolescents aged 7 to 17 years. Hypertension. (2017) 70:897–906. 10.1161/HYPERTENSIONAHA.117.09983 28923902 PMC5722224

[27] Pelikan JM Link T Straßmayr C Waldherr K Alfers T Bøggild H. Measuring comprehensive, general health literacy in the general adult population: the development and validation of the HLS19-Q12 instrument in seventeen countries. Int J Environ Res Public Health. (2022) 19:14129. 10.3390/ijerph192114129 36361025 PMC9659295

[28] Liu R Zhao Q Yu M Chen H Yang X Liu S. Measuring General Health Literacy in Chinese adults: validation of the HLS19-Q12 instrument. BMC Public Health. (2024) 24:1036. 10.1186/s12889-024-17977-1 38622565 PMC11017570

[29] Kowalski KC Crocker PR Donen RM. The physical activity questionnaire for older children (PAQ-C) and adolescents (PAQ-A) manual. Coll Kinesiol Univ Saskatchewan. (2004) 87:1–38.

[30] Liu L Liu Y Zhang T Luo J. Study on the influence of levels of physical activity and socio-economic conditions on body mass index of adolescents. (2024). Int Health. 21:ihae083. 10.1093/inthealth/ihae083 39569471 PMC12212231

[31] Craig C Marshall A Sjostrom M Bauman A Lee P Macfarlane D. International physical activity questionnaire-short form. J Am Coll Health. (2017) 65:492–501.28641040

[32] Amini H Isanejad A Chamani N Movahedi-Fard F Salimi F Moezi M. Physical activity during COVID-19 pandemic in the Iranian population: A brief report. Heliyon. (2020) 6:e05411. 10.1016/j.heliyon.2020.e05411 33163638 PMC7605807

[33] Tran VD Do VV Pham NM Nguyen CT Xuong NT Jancey J. Validity of the international physical activity questionnaire–short form for application in Asian countries: a study in Vietnam. Eval Health Prof. (2020) 43:105–9. 10.1177/0163278718819708 32383410

[34] Frehlich L Friedenreich C Nettel-Aguirre A McCormack G. R. (2018). Test-retest reliability of a modified International Physical Activity Questionnaire (IPAQ) to capture neighbourhood physical activity. J Human Sport Exer. 13:174. 10.14198/jhse.2018.131.17

[35] Regaieg S Charfi N Yaich S Damak J Abid M. The reliability and concurrent validity of a modified version of the international physical activity questionnaire for adolescents (IPAQ-A) in Tunisian overweight and obese youths. Med Princip Pract. (2016) 25:227–32. 10.1159/000442752 26613579 PMC5588365

[36] Gou H Song H Tian Z Liu Y. Prediction models for children/adolescents with obesity/overweight: a systematic review and meta-analysis. Prevent Med. (2024) 179:107823. 10.1016/j.ypmed.2023.107823 38103795

[37] Lee E-Y Shih A-C Tremblay MS. Exploring the world of active play: a comprehensive review of global surveillance and monitoring of active play based on the global matrix data. J Exer Sci Fitness. (2024) 22:254–65. 10.1016/j.jesf.2024.03.008 38577389 PMC10990752

[38] Zhang E Chen J Liu Y Li H Li Y Kuwahara K. Associations between joint lifestyle behaviors and depression among children and adolescents: a large cross-sectional study in China. J Affect Disord. (2024) 352:110–4. 10.1016/j.jad.2024.02.032 38360364

[39] Lee HY Zhou AQ Lee RM Dillon AL. Parents' functional health literacy is associated with children's health outcomes: implications for health practice, policy, and research. Child Youth Serv Rev. (2020) 110:104801. 10.1016/j.childyouth.2020.104801

[40] Paakkari L Kokko S Villberg J Paakkari O Tynjälä J. Health literacy and participation in sports club activities among adolescents. Scand J Public Health. (2017) 45:854–60. 10.1177/1403494817714189 28673131

[41] Solomon-Moore E Toumpakari Z Sebire SJ Thompson JL Lawlor DA Jago R. Roles of mothers and fathers in supporting child physical activity: a cross-sectional mixed-methods study. BMJ Open. (2018) 8:e019732. 10.1136/bmjopen-2017-019732 29358449 PMC5781024

[42] Lloyd AB Lubans DR Plotnikoff RC Collins CE Morgan PJ. Maternal and paternal parenting practices and their influence on children's adiposity, screen-time, diet and physical activity. Appetite. (2014) 79:149–57. 10.1016/j.appet.2014.04.010 24751915

[43] Feldman R. Mutual influences between child emotion regulation and parent–child reciprocity support development across the first 10 years of life: Implications for developmental psychopathology. Dev Psychopathol. (2015) 27:1007–23. 10.1017/S0954579415000656 26439059

[44] Mutz M Albrecht P. Parents' social status and children's daily physical activity: The role of familial socialization and support. J Child Fam Stud. (2017) 26:3026–35. 10.1007/s10826-017-0808-3 29081641 PMC5646135

[45] Morris AS Silk JS Steinberg L Myers SS Robinson LR. The role of the family context in the development of emotion regulation. Soc Dev. (2007) 16:361–88. 10.1111/j.1467-9507.2007.00389.x 19756175 PMC2743505

[46] Kaur J Upendra S Barde S. Inhaling hazards, exhaling insights: a systematic review unveiling the silent health impacts of secondhand smoke pollution on children and adolescents. Int J Environ Health Res. (2024) 34:4059–4073. 10.1080/09603123.2024.2337837 38576330

[47] Li M Ren Y. Relationship among physical exercise, social support and sense of coherence in rural left-behind children. J Psychiatr Res. (2024) 169:1–6. 10.1016/j.jpsychires.2023.11.010 37995496

[48] Diao Y Wang L Chen S Barnett LM Mazzoli E Essiet IA. validity of the physical literacy in children questionnaire in children aged 4 to 12. BMC Public Health. (2024) 24:869. 10.1186/s12889-024-18343-x 38515090 PMC10956319

[49] Liang Y Rascle O Hanel PH Yang J Souchon N. Values and physical activity among sports science students in France and China: a transcultural analysis. Front Psychol. (2024) 14:1304019. 10.3389/fpsyg.2023.1304019 38239479 PMC10794636

[50] Dong M Niu M Jiang Z Choi Y Li N. A study on the impact of sports participation support on the level of sports participation of urban junior high school girls in China. Front Psychol. (2025) 15:1539415. 10.3389/fpsyg.2024.1539415 39980880 PMC11841449

[51] Hunter SK Angadi SS Bhargava A Harper J Hirschberg AL Levine BD. The biological basis of sex differences in athletic performance: consensus statement for the American College of Sports Medicine. Transl J Am Coll Sports Med. (2023) 8:1–33. 10.1249/TJX.0000000000000236 37772882

[52] Ge X Zhang E Liu Y Li H Hu F Chen J. Factors associated with out-of-school physical activity among Chinese children and adolescents: a stratified cross-sectional study. Prevent Med. (2024) 184:107985. 10.1016/j.ypmed.2024.107985 38705485

[53] Liu Y Tang Y Cao Z-B Zhuang J Zhu Z Wu X-P. Results from the China 2018 Report Card on physical activity for children and youth. J Exer Sci Fitness. (2018) 17:3. 10.1016/j.jesf.2018.10.002 30662507 PMC6323175

[54] Chen J Bai Y Ni W. Reasons and promotion strategies of physical activity constraints in obese/overweight children and adolescents. Sports Med Health Sci. (2024) 6:25–36. 10.1016/j.smhs.2023.10.004 38463665 PMC10918361

