# Supplementary material for: Maternal health literacy plays a greater role than paternal health literacy in adolescent physical activity in China: a cross-sectional study
Source: Front Public Health. 2025 May 19;13:1585615. doi: 10.3389/fpubh.2025.1585615 (PMC12127411; doi:10.3389/fpubh.2025.1585615)
Supplement: Supplementary file 1 [file Table_1.docx]

**题目及答案选项：**

**"在健康相关话题上，获取易懂、可靠和有用的信息并不总是容易的。以下问题旨在了 在处理健康信息时，哪些任务较为容易或较为困难。请根据从非常容易到非常困难的尺度，告诉我们您认为以下任务的难易程度：**

1. … 找到生病时获得专业帮助的地方，您觉得有多容易？
2. … 理解有关在医疗紧急情况下该做什么的信息，您觉得有多容易？
3. … 判断不同治疗方案的优缺点，您觉得有多容易？
4. … 按照医生或药剂师的建议采取行动，您觉得有多容易？
5. … 找到如何应对心理健康问题的信息，您觉得有多容易？
6. … 理解有关推荐健康筛查或检查的信息，您觉得有多容易？
7. … 判断有关不健康习惯（如吸烟、缺乏运动或饮酒过多）是否可靠，您觉得有多容易？
8. … 根据大众媒体提供的信息决定如何保护自己免受疾病的侵害，您觉得有多容易？
9. … 找到关于健康生活方式（如体育锻炼、健康饮食或营养）的信息，您觉得有多容易？
10. … 理解来自家庭或朋友的有关健康建议，您觉得有多容易？
11. … 判断住房条件如何影响您的健康和福祉，您觉得有多容易？
12. … 做出改善健康和福祉的决策，您觉得有多容易？**

**答案选项：**

- 非常容易
- 容易
- 难
- 非常难
- 不知道/拒绝（自发）
