# Supplementary material for: Maternal health literacy plays a greater role than paternal health literacy in adolescent physical activity in China: a cross-sectional study
Source: Front Public Health. 2025 May 19;13:1585615. doi: 10.3389/fpubh.2025.1585615 (PMC12127411; doi:10.3389/fpubh.2025.1585615)
Supplement: Supplementary file 2 [file Table_2.docx]

HLS19-Q12

“It is not always easy to get understandable, reliable, and useful information on health-related topics. With the following questions we would like to find out which tasks related to handling health information are more or less easy or difficult. On a scale from very easy to very difficult, how easy would you say it is …

1. … to find out where to get professional help when you are ill?

2. … to understand information about what to do in a medical emergency?

3. … to judge the advantages and disadvantages of different treatment options?

4. … to act on advice from your doctor or pharmacist?

5. … to find information on how to handle mental health problems?

6. … to understand information about recommended health screenings or examinations?

7. … to judge if information on unhealthy habits, such as smoking, low physical activity or drinking too much alcohol, are reliable?

8. … to decide how you can protect yourself from illness using information from the mass media?

9. … to find information on healthy lifestyles such as physical exercise, healthy food or nutrition?

10. … to understand advice concerning your health from family or friends?

11. ... to judge how your housing conditions may affect your health and well-being?

12. … to make decisions to improve your health and well-being?”

Response categories: “Very easy”, “Easy”, “Difficult”, “Very difficult”, “Don’t Know / Refusal (SPONTANEOUS)”
