# Supplementary material for: Maternal health literacy plays a greater role than paternal health literacy in adolescent physical activity in China: a cross-sectional study
Source: Front Public Health. 2025 May 19;13:1585615. doi: 10.3389/fpubh.2025.1585615 (PMC12127411; doi:10.3389/fpubh.2025.1585615)
Supplement: Supplementary file 4 [file Table_4.docx]

**国际身体活动问卷（IPAQ）**

**过去七天简短版**

我们对人们日常生活中所做的身体活动类型感兴趣。以下问题将询问您在过去七天内从事身体活动的时间。即使您不认为自己是一个活跃的人，也请回答每一个问题。请考虑您在工作中、家务和庭院工作、从一个地方到另一个地方的交通，以及休闲、锻炼或运动时所做的活动。

请考虑您在过去七天内所做的所有剧烈活动。剧烈身体活动是指那些需要较大体力付出并让您呼吸比平常更加急促的活动。请只考虑那些每次至少做了10分钟的身体活动。

1. 在过去七天内，您在多少天进行过剧烈的身体活动，如举重、挖掘、健美操或快速骑行？
   _____ 每周几天
   没有剧烈身体活动 —— 跳到问题3
2. 在那些天里，您通常每次做剧烈身体活动的时间是多少？
   _____ 每天几个小时
   _____ 每天几分钟
   不知道/不确定

请考虑您在过去七天内做的所有中等强度的活动。中等强度活动是指那些需要中等体力付出并让您呼吸比平常稍微急促的活动。请只考虑那些每次至少做了10分钟的身体活动。

1. 在过去七天内，您在多少天进行过中等强度的身体活动，如搬运轻物、骑自行车以正常速度或打双打网球？不包括走路。
   _____ 每周几天
   没有中等强度身体活动 —— 跳到问题5
2. 在那些天里，您通常每次做中等强度身体活动的时间是多少？
   _____ 每天几个小时
   _____ 每天几分钟
   不知道/不确定

请考虑您在过去七天内的走路时间。这包括工作和家里走路、从一个地方到另一个地方的步行，以及您纯粹为了休闲、运动、锻炼或娱乐所做的任何其他步行。

1. 在过去七天内，您在多少天走路至少10分钟一次？
   _____ 每周几天
   没有走路 —— 跳到问题7
2. 在那些天里，您通常每次走路的时间是多少？
   _____ 每天几个小时
   _____ 每天几分钟
   不知道/不确定

最后一个问题是关于您在过去七天内平日里坐着的时间。包括在工作、家里、做作业和休闲时间坐着的时间。这可能包括坐在桌前、拜访朋友、阅读，或坐着或躺下看电视。

1. 在过去七天内，您在工作日里每天坐着的时间是多少？
   _____ 每天几个小时
   _____ 每天几分钟
   不知道/不确定
